# Copolymers of Vinyl-Containing Benzoxazine with Vinyl Monomers as Precursors for High Performance Thermosets

**DOI:** 10.3390/molecules20046488

**Published:** 2015-04-10

**Authors:** Tsutomu Takeichi, Soulideth Thongpradith, Takehiro Kawauchi

**Affiliations:** Department of Environmental and Life Sciences, Toyohashi University of Technology, Tempaku-cho, Toyohashi 441-8580, Japan; E-Mails: detto_me@yahoo.com (S.T.); kawauchi.t.aa@m.titech.ac.jp (T.K.)

**Keywords:** benzoxazine, vinyl monomer, radical polymerization, high molecular weight benzoxazine, ring-opening polymerization

## Abstract

A benzoxazine containing a vinyl group (P-4va) was prepared by the reaction of phenol, 4-vinylaniline, and paraformaldehyde. A differential scanning calorimetry (DSC) study revealed that ring-opening polymerization of the benzoxazine and chain polymerization of the vinyl group occurred in the same temperature range. When 2,2'-azobisisobutyronitrile was added as a radical initiator to P-4va, however, only the vinyl groups were polymerized at lower temperature, giving oligo(P-4va) that contains pendent benzoxazine units. Radical copolymerization of P-4va with various vinyl monomers such as styrene, methyl methacrylate (MMA), and *n*-butyl acrylate (BuA) was examined. The chemical structure of the copolymers was confirmed by FT-IR and ^1^H-NMR to be one of polyolefins bearing benzoxazine units as the pendant groups. The weight-average molecular weights of the copolymers determined by size exclusion chromatography were to be in the range of 1900–51,500 depending on the comonomers. DSC of the copolymers showed that the maxima of the exothermic peaks corresponding to the ring-opening polymerization of the pendent benzoxazine units were observed in the temperature range of 229–250 °C. Thermal cure up to 240 °C of the copolymer films afforded homogenous transparent films with improved thermal properties. Tough cured film was obtained by the copolymerization with MMA, while a tough and flexible film was obtained by the copolymerization with BuA.

## 1. Introduction

**Scheme 1:**
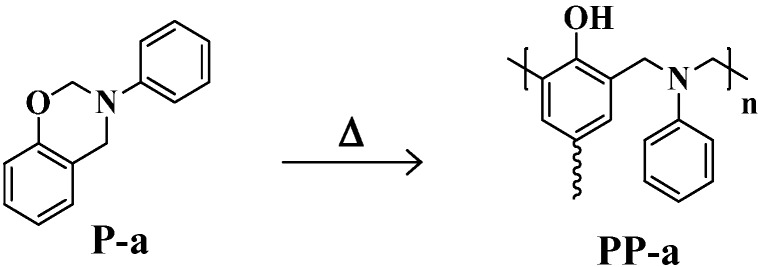
Ring-opening polymerization of P-a.

Polybenzoxazines (PBZs) have been developed as a novel type of high performance phenolic resin [1,2,3,4,5,6,7]. PBZs are obtained by the ring-opening polymerization of cyclic monomers, benzoxazines (BZs). The chemical structures of a typical mono-functional BZ, P-a, and the thermally cured PBZ, PP-a, are shown in Scheme 1. PBZs offer molecular design flexibility because of the wide availability of the starting phenols and amines. PBZs possess characteristics found in the traditional phenolic resins, such as excellent thermal properties, good flame retardancy, and good electrical properties. PBZs also offer unique characteristics that are not found in the traditional phenolic resins, including releasing no by-products, no need of strong acid or base catalyst as well as excellent dimensional stability [8,9,10], low viscosity [11], low dielectric constant [12], and low surface free energy [13]. Therefore, PBZs overcome most of the drawbacks of the traditional phenolic resins, and have been attracting significant attention for industrial applications.

However, PBZs have some drawbacks, such as the high curing temperature necessary for the ring-opening polymerization (*ca.* 200 °C) and brittleness, as is common for thermosets. Also, the thermal properties are not sufficient enough to withstand in harsh conditions. In order to overcome the drawbacks, various approaches have been attempted. One approach is the modification of BZ monomers. For example, introducing cross-linkable units gives cured resins with increased cross-link density, leading to the much higher glass transition temperature (*T*_g_) [14,15,16,17]. High molecular weight BZs that have BZ moieties in the backbone or in the pendent part have been successful in obtaining PBZs with enhanced thermal and mechanical properties [18,19,20].

Another approach is alloying PBZ with rubbery or thermoplastic polymers, which has been shown to be one of the most successful approaches that can significantly enhance the performance of the thermosets [21,22,23,24,25,26,27,28,29,30,31,32,33,34,35,36,37,38]. For instance, the alloying with liquid rubbers can improve the toughness of PBZ [33,34,35]. Introduction of hydrophenylmaleimide further improved the performance of rubber-modified PBZ [36]. For high-performance application, the thermally curable BZ was introduced into the main-chain of polystyrene to form macromonomers [37]. Modification of typical BZ with methyl methacrylate (MMA) through free radical polymerization in the presence of photosensitizers has also been reported [38]. In the previous paper [39], we reported the preparation of novel BZs having vinyl groups (P-4va), aiming to enhance the thermal properties of PBZ. The thermally cured PBZ of P-4va showed enhanced *T_g_* up to 244 °C compared to the *T*_g_ (*ca.* 175 °C) of the typical PBZ without vinyl group (PP-a). The significant enhancement in *T_g_* is due to the increased cross-link density due to the chain polymerization of the vinyl group in addition to the ring-opening polymerization of BZ. In this study, taking advantage of the copolymerizable double bond of P-4va, P-4va was copolymerized with various vinyl monomers, affording polyolefins containing pendant BZs, which were then thermally cured by the ring-opening polymerization of BZ units, with the expectation of thus developing a novel approach to obtain high performance PBZs.

## 2. Results and Discussion 

### 2.1. Radical Homopolymerization of P-4va

**Scheme 2:**
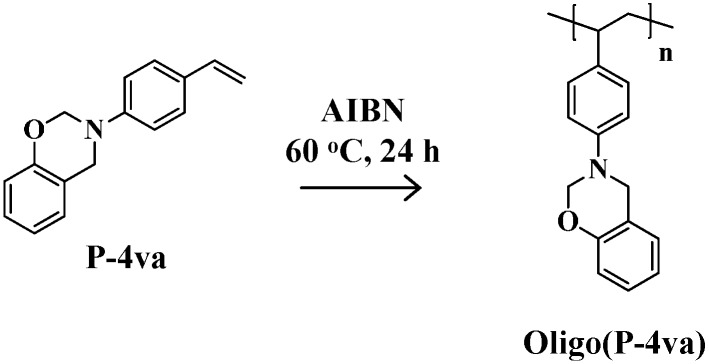
Radical polymerization of P-4va.

We reported in the previous paper that thermal cure of P-4va gives a cross-linked polymer (PP-4va) produced by the chain polymerization of the vinyl group and the ring-opening polymerization of BZ units in the same temperature range [39]. In this present study, first of all, we investigated the radical polymerizability of the vinyl groups of P-4va in the presence of a radical initiator, AIBN. We expected that linear polymers containing BZ units as pendant groups could be obtained by the radical polymerization as shown in Scheme 2. We abbreviated the polymeric product as oligo(P-4va) because of the moderate molecular weight, as will be reported later.

**Figure 1:**
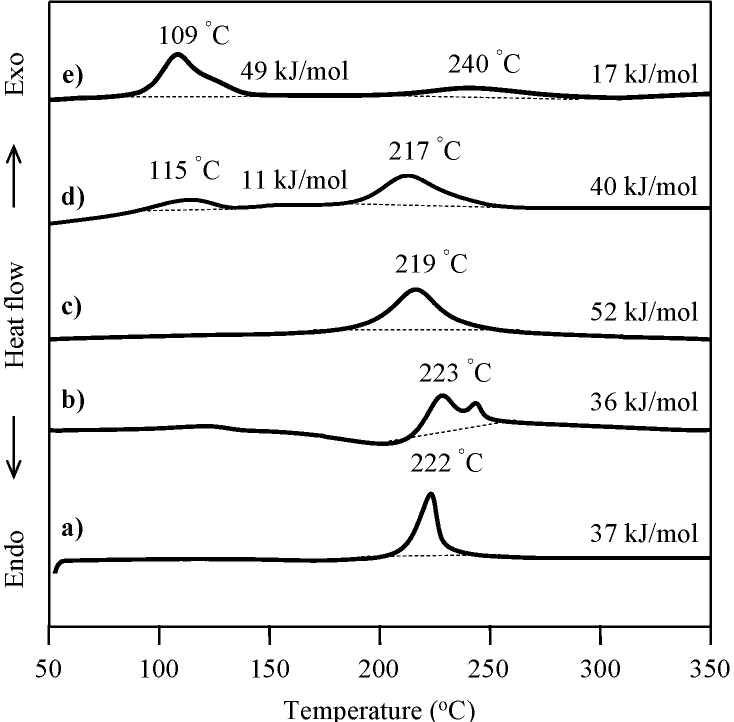
DSC thermograms of various BZs. P-a (a), P-a/AIBN(10 mol %) (b), P-4va (c), P-4va/AIBN (3 mol %) (d), and P-4va/AIBN (10 mol %) (e).

The effect of radical initiator, AIBN, on the radical polymerization of P-4va was monitored by DSC. In the case of P-a, a typical BZ without vinyl group, as shown in Figure 1a,b, the addition of AIBN did not cause any significant change in the exotherm. On the other hand, in the case of P-4va, by adding 3 mol % of AIBN, an exotherm appeared at much lower temperature range in addition to the exotherm due to the ring-opening polymerization of BZs (Figure 1c,d). Further addition of AIBN further lowered the temperature (Figure 1e). The exotherm at a lower temperature range is considered to be due to the free radical polymerization of the double bond of P-4va.

Radical polymerization of P-4va with AIBN was carried out in benzene at 60 °C for 24 h. MeOH-insoluble material, oligo(P-4va), was obtained as pale pink powder in 64% yield. The MeOH-insoluble material was dissolved into chloroform, and subjected to SEC measurement.

**Figure 2:**
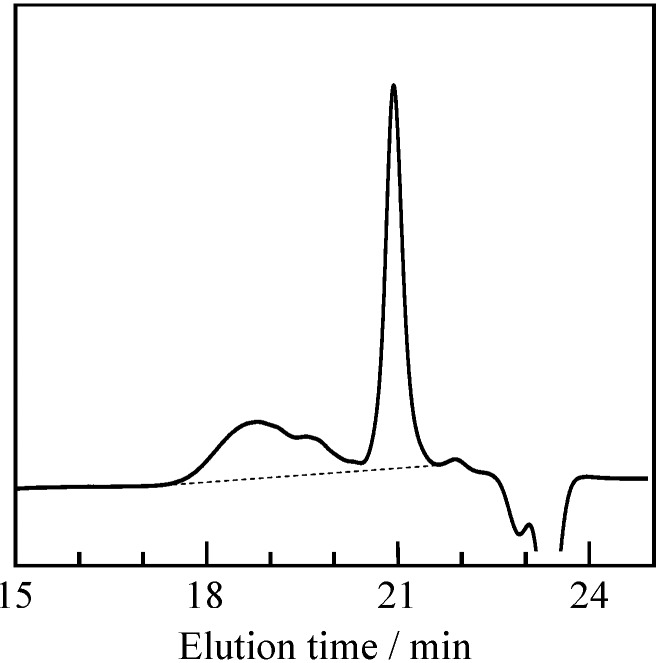
SEC profile of oligo(P-4va).

The SEC profile revealed a multimodal molecular weight distribution (Figure 2). The weight-average molecular weight (*M*_w_) and molecular weight distribution (*M*_w_/*M*_n_) of the oligomer was calculated to be 1070 and 1.65, respectively, indicating the low radical polymerizability of P-4va. Bulkiness of the benzoxazine pendant groups might affect the polymerizability.

**Figure 3:**
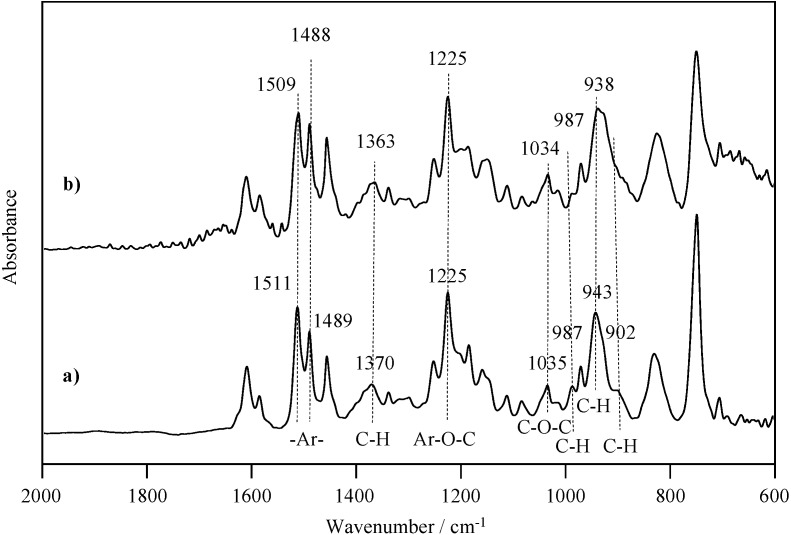
IR spectra of P-4va (a) and oligo(P-4va) (b).

IR spectra of P-4va and oligo(P-4va) are shown in Figure 3. P-4va shows vinyl group absorptions (1489 and 1511 cm^−1^, C=C and –Ar–, di-substituted benzene) and characteristic absorptions at 943 cm^−1^ (C-H, cyclic vibration mode), 1035 cm^−1^ (C-O-C, symmetric stretching), 1225 cm^−1^ (C-O-C, antisymmetric stretching), 1370 cm^−1^ (C-H, vibration mode of CH_2_), and 987 cm^−1^ (C-H, out of plane deformation vibration). In the IR spectrum of oligo(P-4va), the C-H out of plane deformation vibration of the vinyl group at 902 cm^−1^ disappeared, and the absorption band at 987 cm^−1^ almost disappeared, indicating that the radical polymerization of the P-4va proceeded through the chain polymerization of the vinyl group. 

**Figure 4:**
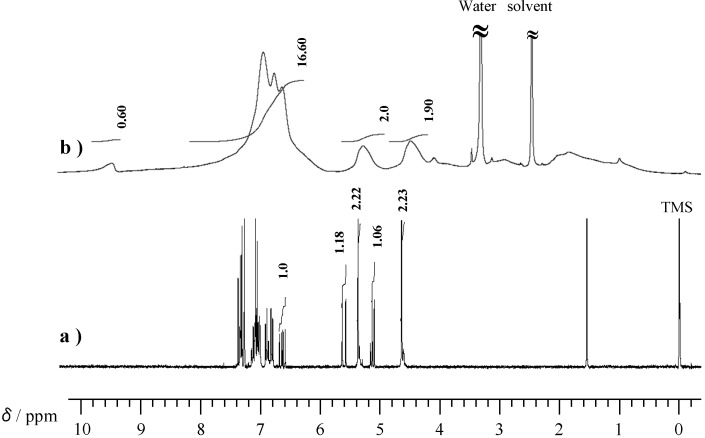
^1^H-NMR of P-4va (a) and oligo(P-4va) (b).

Figure 4 shows ^1^H-NMR spectra of P-4va and oligo(P-4va). The oligo(P-4va) did not show olefinic proton signals, confirming that the radical polymerization of the vinyl group of P-4va had ocurred. Moreover, typical protons of BZ were observed at 4.53 (Ar-*CH_2_*-N) and 5.32 ppm (O-*CH_2_*-N). The integral ratio agreed with the chemical structure of oligo(P-4va). These results indicate that the radical polymerization gave a linear polyolefin having pendant BZ units.

### 2.2. Radical Copolymerization of P-4va with St, MMA, and BuA

**Scheme 3:**
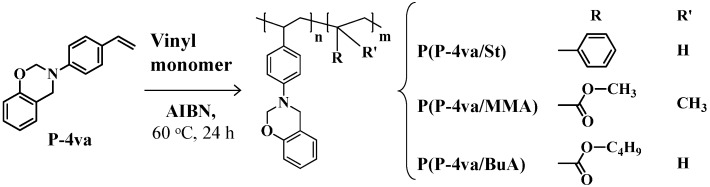
Radical copolymerization of P-4va with various vinyl monomers.

Radical copolymerization of P-4va with various vinyl monomers was carried out in the presence of AIBN at 60 °C for 24 h in benzene (Scheme 3). The results are summarized in Table 1.

**Figure 5:**
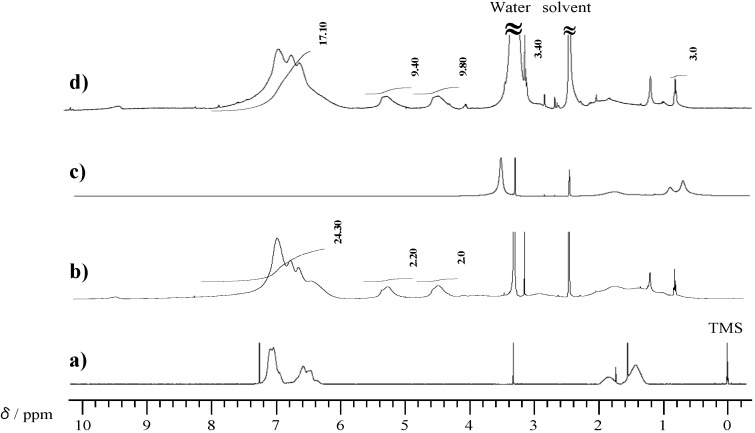
^1^H-NMR spectra of PSt (a), P(P-4va/St) (b), PMMA (c), and P(P-4va/MMA) (d). The spectra were measured in DMSO-*d*_6_, except for PSt. The spectrum of PSt were measured in CDCl_3_.

Copolymerization of P-4va with St at 1:1 molar ratio gave a polymeric product with 77 mol % of St composition (P(P-4va/St)) in 60% yield. The product was soluble in polar solvents such as chloroform, THF, DMF and DMSO, as well as non-polar solvents such as benzene. The *M*_w_ of the P(P-4va/St) copolymer was evaluated to be 8300 by SEC (Figure S1 in the Supporting Information), which is much higher than that of oligo(P-4va) (*M*_w_ = 1070). Similarly, copolymerization of P-4va and MMA in 9:1 molar ratio with AIBN was carried out in benzene at 60 °C for 24 h. As shown in Table 1, the copolymer with 17 mol % of MMA component (P(P-4va/MMA)) was obtained in 57% yield, whose *M*_w_ was 1900. ^1^H-NMR spectra of the copolymers are shown in Figure 5. In the spectrum of P(P-4va/St), characteristic signals of BZ units were observed at 4.5 and 5.3 ppm (methylene protons of the BZ ring), confirming that the BZ unit was introduced into the polystyrene chain as the pendant (Figure 5b). Same was true for the P(P-4va/MMA) copolymer (Figure 5d).

**Table 1:** Radical copolymerization of P-4va with vinyl monomers in benzene at 60 °C for 24 h.

Code	Monomer feed ^a^ (mol %)		Yield ^b^ (%)	Composition ^c^ (mol %)		*M*_w_^d^	*M*_w_/*M*_n_^d^
	P-4va	Comonomer		f_1_	f_2_		
oligo(P-4va)	100	-	64	100	-	1070	1.65
PSt	-	100	52	-	100	33,100	1.84
PMMA	-	100	73	-	100	47,800	1.80
PBuA	-	100	80	-	100	98,800	5.40
P(P-4va/St)	50	50	60	23	77	8300	2.96
P(P-4va/MMA)	90	10	57	83	17	1900	1.81
P(P-4va/BuA)	90	10	98	86	14	3400	2.43
P(P-4va/BuA)	70	30	92	67	33	13,800	5.11
P(P-4va/BuA)	50	50	86	50	50	14,000	1.28
P(P-4va/BuA)	30	70	83	42	58	16,400	1.26
P(P-4va/BuA)	10	90	64	37	63	51,500	1.84

^a^ [AIBN] = 0.1 mmol, [P-4va + comonomer] = 10 mmol, benzene 2 mL. ^b^ Products after purification. ^c^ Determined by ^1^H-NMR. ^d^ Determined by SEC. See Figures S1 and S2 in Supporting Information.

**Figure 6:**
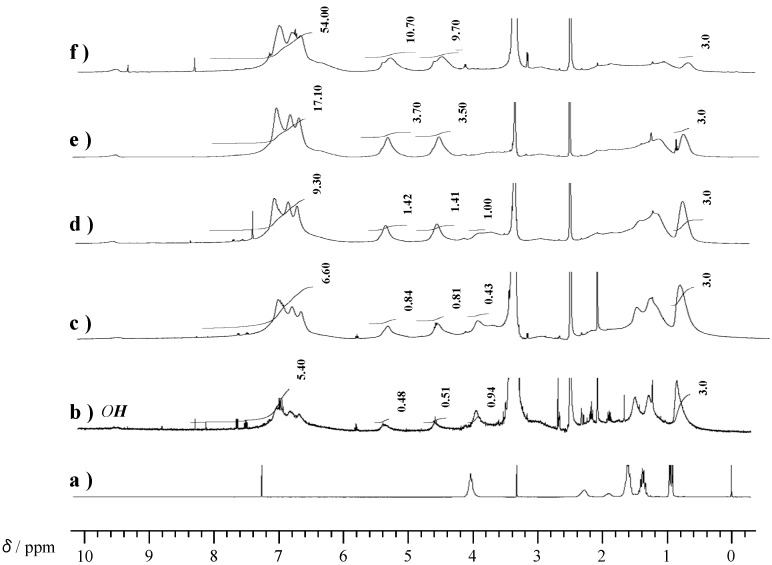
^1^H-NMR spectra of PBuA (a), and P(P-4va/BuA) with 63 (b), 58 (c), 50 (d), 33 (e), and 14 (f) mol % of BuA composition prepared at 90, 70, 50, 30, and 10 mol % of BuA feed ratio, respectively.

Next, copolymerization of P-4va and BuA with various composition ratios was performed. With increasing BuA composition, the yield of the copolymer was decreased, but the *M*_w_ of the copolymers gradually increased (Table 1); the *M*_w_s of the copolymers prepared with 10, 30, 50, 70, and 90 mol % of BuA composition were 3400, 13,800, 14,000, 16,400, and 51,500, respectively (Figure S2 in the Supporting Information). Figure 6 shows the ^1^H-NMR spectra of the copolymers. Methyl signals corresponding to the BuA unit were observed at around 1 ppm. It was confirmed that characteristic signals at 4.5 and 5.3 ppm due to the BZ unit increased with increasing P-4va composition on the basis of the intensity ratio with the methyl signal. Although P-4va showed low radical polymerizability as evidenced by homopolymerization with AIBN, the P-4va units were successfully introduced into the polymer chain by copolymerization with common vinyl monomers.

### 2.3. Thermal Curing Behavior of Oligo(P-4va) and Copolymers

**Scheme 4:**
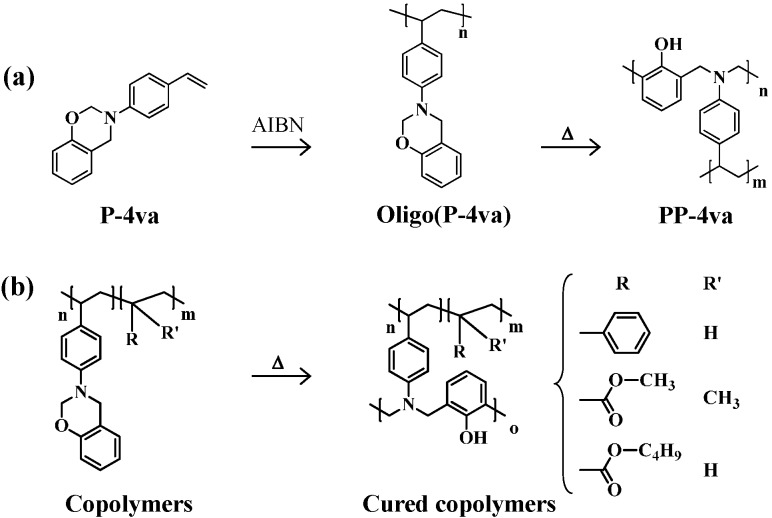
Thermal cure of oligo(P-4va) (**a**) and copolymers (**b**).

The chemical structures of PBZs derived from oligo(P-4va) and the copolymers after thermal ring-opening polymerization of the BZ units are shown in Scheme 4.

**Figure 7:**
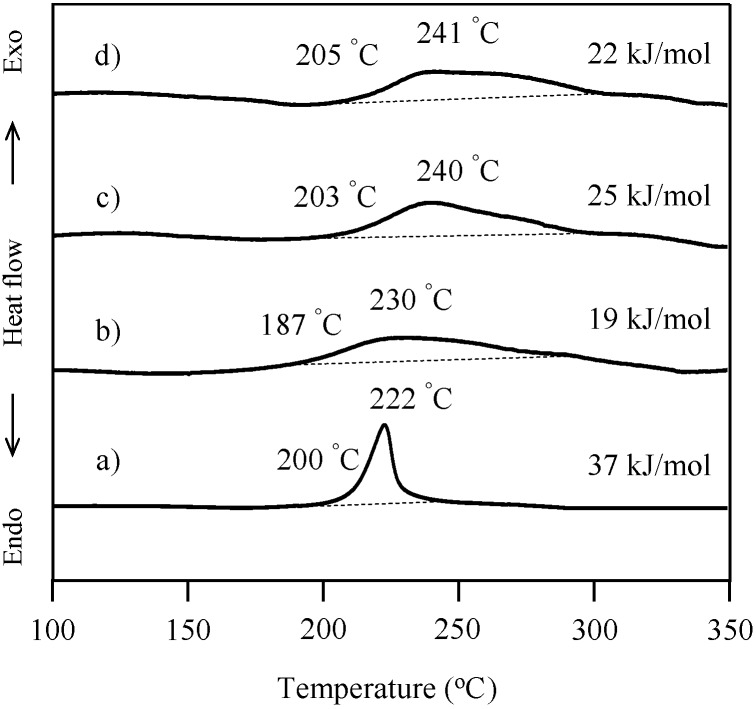
DSC thermogrmas of P-a (a), oligo(P-4va) (b), P(P-4va/St) (c), and P(P-4va/MMA) (d). The exothermic amounts were calculated on the basis of the benzoxazine monomeric unit.

The cure behavior of oligo(P-4va) and copolymers was monitored by DSC. The DSC thermogram of typical BZ, P-a, exhibited the onset of a sharp exotherm at 200 °C with the maximum at 222 °C, and the exotherm due to the ring-opening polymerization of BZ was measured at 37 kJ/mol (Figure 7a). Oligo(P-4va) also gave a broad exotherm with onset at 187 °C with a maximum at 235 °C and 19 kJ/mol as exotherm heat, showing that the ring-opening polymerization of the pendant BZ unit occurred during the DSC measurement. The ring-opening polymerization behavior of the pendent BZ components in each copolymer, P(P-4va/St), P(P-4va/MMA), and P(P-4va/BuA) was also observed in their DSC thermograms as the onset of exotherms at 203, 205, and 192 °C, respectively, with the corresponding maxima at between 240, 241, and 235 °C, respectively. The measured exotherms were normalized on the basis of the benzoxazine monomeric unit. The values of the copolymers were in the range of 22–31 kJ/mol, which is almost same as the size of the exotherm of typical BZ, P-a, or oligo(P-4va) without the vinyl monomer components.

**Figure 8:**
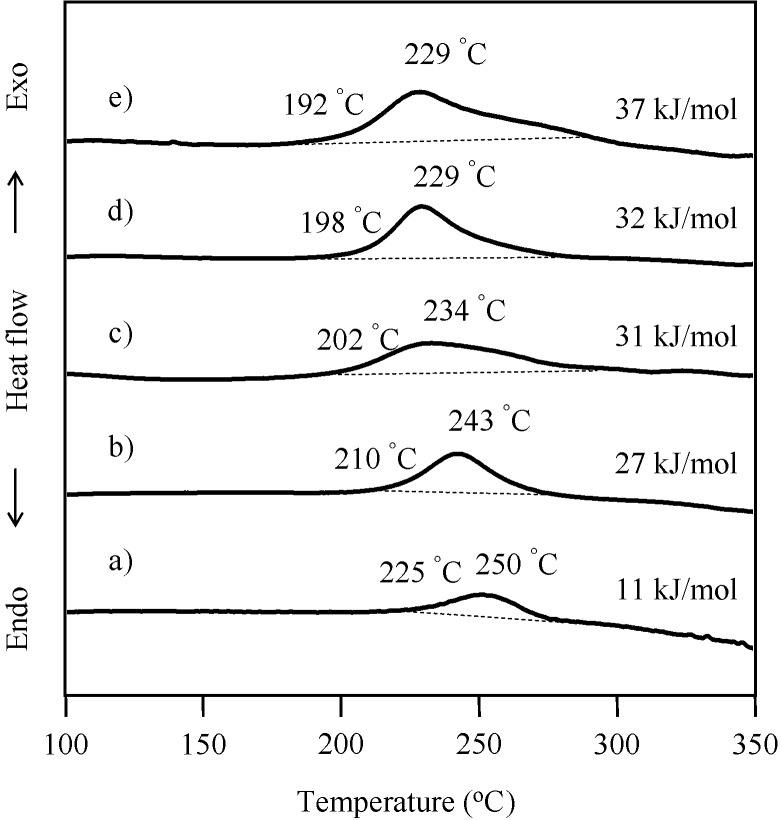
DSC curves of P(P-4va/BuA) with 63 (a), 58 (b), 50 (c), 33 (d), and 14 (e) mol % of BuA composition. The exothermic amounts were calculated on the basis of the benzoxazine monomeric unit.

The ring-opening polymerization of the BZ unit in the P(P-4va/BuA) copolymers with various BuA compositions was also confirmed by DSC measurement as shown in Figure 8. The exotherm onsets were in the range of 192–225 °C with the maxima observed in the range of 229–250 °C.

### 2.4. Thermal Cure of Oligo(P-4va) and the Copolymers 

**Figure 9:**
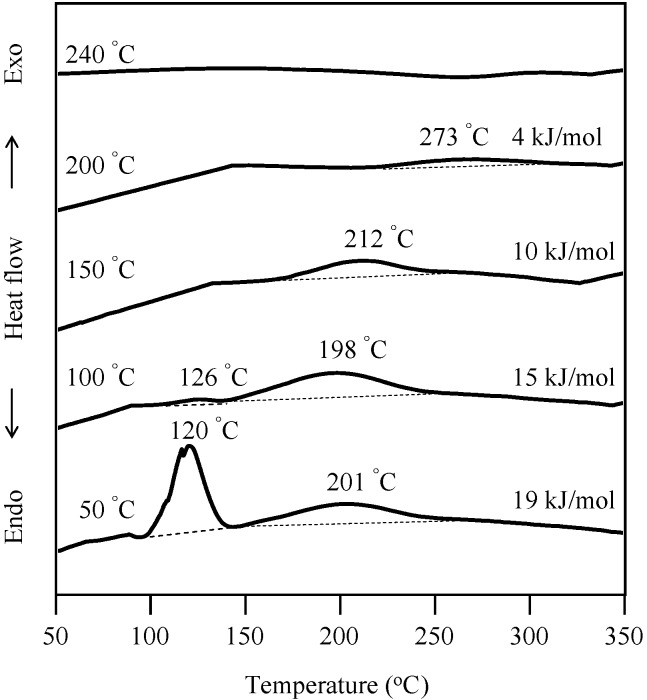
DSC thermograms of oligo(P-4va) after each cure stage.

Cast films of oligo(P-4va) and the copolymers on glass substrates were dried at 50 °C for 12 h and then cured at 100, 150, 200 and 240 °C for 1 h each to obtain free-standing corresponding PBZ films. The cure progress of oligo(P-4va) was followed by DSC as shown in Figure 9. The exothermic peak corresponding to the ring-opening polymerization of BZ gradually decreased with the increase of curing temperature, and completely disappeared after cure at 240 °C, indicating the final cure temperature was enough to finish the polymerization of the BZ units.

### 2.5. Properties of the Thermally Cured Films

**Figure 10:**
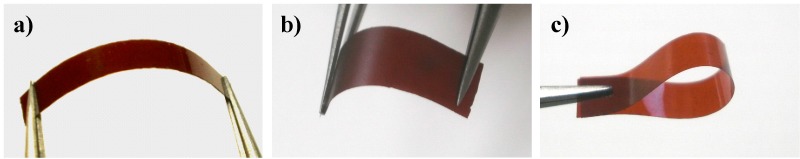
Appearance of cured films of PP-4va (**a**), P(P-4va/MMA) (**b**), and P(P-4va/BuA) with 33 mol % of BuA composition (**c**).

We previously reported that thermal cure of P-4va gave a free-standing film (PP-4va) [39]. Figure 10a shows appearance of the PP-4va films, which were tough enough to measure their mechanical properties. Interestingly, the cured oligo(P-4va) film was very brittle, so we could not measure its mechanical properties, indicating the polymerization procedure strongly affected the network structure. The P(P-4va/St) copolymers also gave a very brittle cured film. On the other hand, the cured P(P-4va/MMA) film was tough enough for mechanical measurements (Figure 10b). Moreover, flexible and tough films were obtained from the P(P-4va/BuA) copolymers with 33–58 mol % of BuA composition (Figure 10c). Additionally the mechanical properties of the the cured film of P(P-4va/BuA) with 63 mol % of BuA composition could not be measured because the nature of the rubber component led to a very rubbery film.

**Figure 11:**
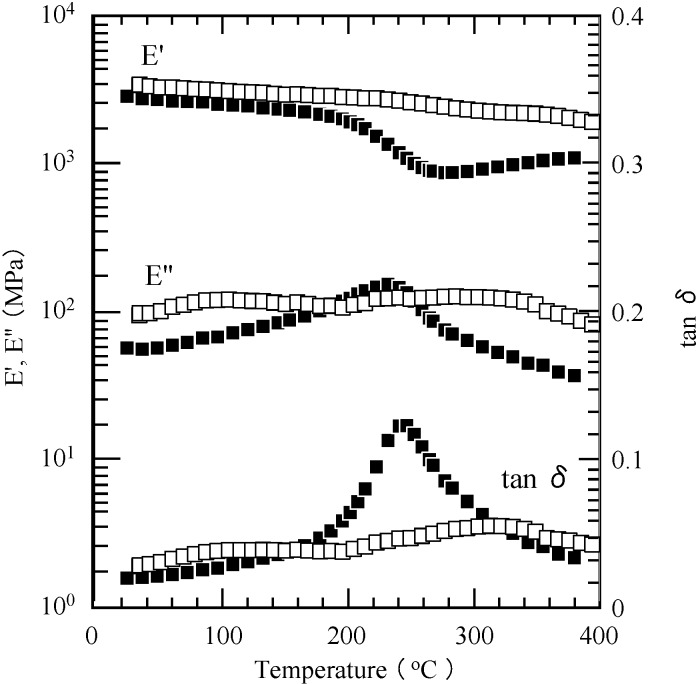
Viscoelastic properties of PP-4va (■), and P(P-4va/MMA) (□).

The thermal properties of the cured films of PP-4va and P(P-4va/MMA) were characterized by DMA (Figure 11). The results are summarized in Table 2. 

**Table 2:** Thermal properties of the cured films.

Code	Composition ^a^ (mol %)		*T*_g_ (°C)			*T*_5_^b^ (°C)	*T*_10_^b^ (°C)	Char yield at 850 °C ^b^ (%)
			DMA					
	f1	f2	E''	tan δ	DSC			
PP-4va	100	-	230 ^c^	244 ^c^	-	355 ^c^	378 ^c^	48 ^c^
oligo(P-4va)	100	-	-	-	-	364	394	53
PSt	-	100	-	-	70	346	372	0
PMMA	-	100	64	64	73	332	343	0
PBuA	-	100	-	-	−62	328	346	0.7
P(P-4va/St)	23	77	-	-	-	345	378	45
P(P-4va/MMA)	83	17	286	307	-	346	373	44
P(P-4va/BuA)	86	14	-	-	-	352	385	44
P(P-4va/BuA)	67	33	238	262	-	349	380	43
P(P-4va/BuA)	50	50	253	283	42	352	380	49
P(P-4va/BuA)	42	58	235	277	−12	339	363	35
P(P-4va/BuA)	37	63	-	-	−55	346	360	12

^a^ Determined by ^1^H-NMR. ^b^ Determined by TGA. ^c^ Reference [39].

The cured PP-4va film revealed *T_g_* at 230 °C from E'' and 244 °C from tan δ, which are higher than those of the typical PBZ without vinyl groups (*ca.* 175 °C) due to the chain polymerization of the vinyl group and the ring-opening polymerization of BZ [39]. Surprisingly, the cured film of P(P-4va/MMA) with 17 mol % of MMA composition exhibited a significantly improved *T_g_*, at 286 °C from E'' and 307 °C from tan δ. This strengthening *T_g_* resulted from the in-chain segmental motion interaction of the long chain amine between BZ backbones and MMA components in the copolymer system. Furthermore, the introduction of PMMA into the network structure of PBZ enhanced the storage modulus (E') at 40 °C, which was increased from 2.8 to 3.4 GPa.

**Figure 12:**
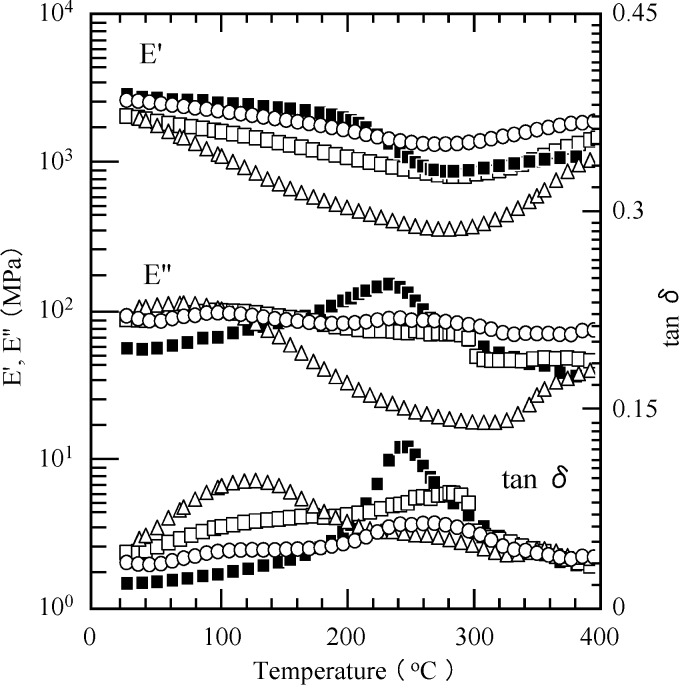
Viscoelastic properties of cured films of PP-4va (■), and P(P-4va/BuA) with 33 (○), 50 (□), and 58 (∆) mol % of BuA composition.

The thermomechanical properties of the cured P(P-4va/BuA) films with various composition were also investigated by DMA measurements (Figure 12). The *T_g_* of pure PBuA was estimated to be *ca.* −60 °C by DSC.

The cured film of P(P-4va/BuA) with 50 mol % of BuA composition exhibited a *T_g_* as high as 253 °C from E'', and 283 °C from tan δ. Similar to the case of PMMA, the various BuA compositions seemed to affect the thermal transition of PBZ. PBuA also significantly improved the toughness of the PBZ, depending on the composition of PBuA moieties, however, the increase of PBuA component leads to a decrease of the storage modulus, as can be seen from the cured film with 53 mol % of BuA composition. 

**Figure 13:**
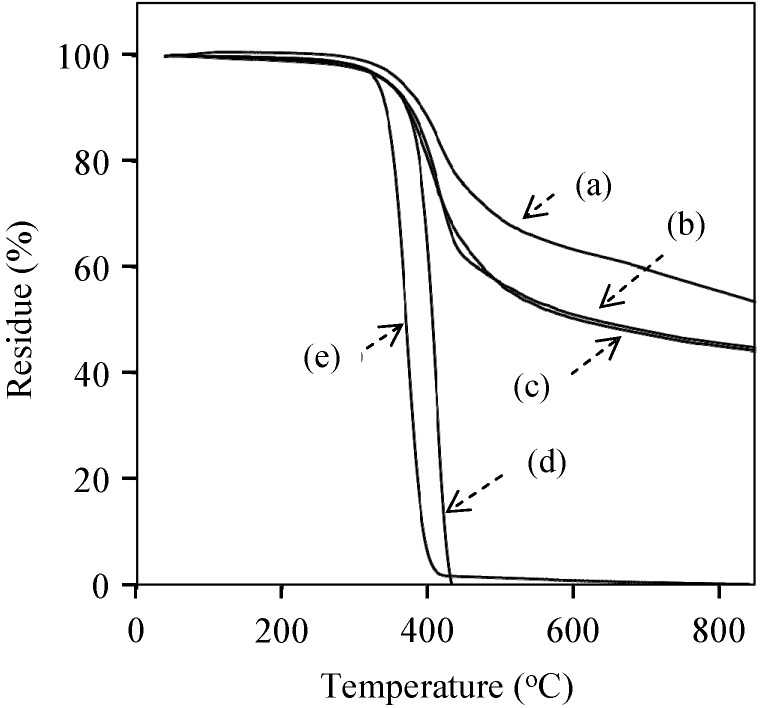
TGA curves of the cured films of oligo(P-4va) (a), P(P-4va/St) (b), and P(P-4va/MMA) (c), and PSt (d) and PMMA (e).

Thermal stability of the cured films was investigated by TGA under an argon atmosphere (Figure 13 and Table 2). The 5% and 10% weight loss temperatures (*T*_5_ and *T*_10_) of the cured film of oligo(P-4va) were estimated to be 364 and 394 °C, respectively. The char yield at 850 °C was as high as 53%. Both PSt and PMMA films decomposed completely at *ca.* 400 °C. Interestingly, in spite of the high St content (77 mol %), P(P-4va/St) shows *T*_5_ and *T*_10_ at 345 °C and 378 °C, respectively, with as high as 45% of char yield at 850 °C. P(P-4va/MMA) also revealed high thermal stability, which is almost same as P(P-4va/St). From the TGA measurement, it can be concluded that the thermal stability of the cured films from the copolymers was almost similar to that of the cured oligo(P-4va) film. 

**Figure 14:**
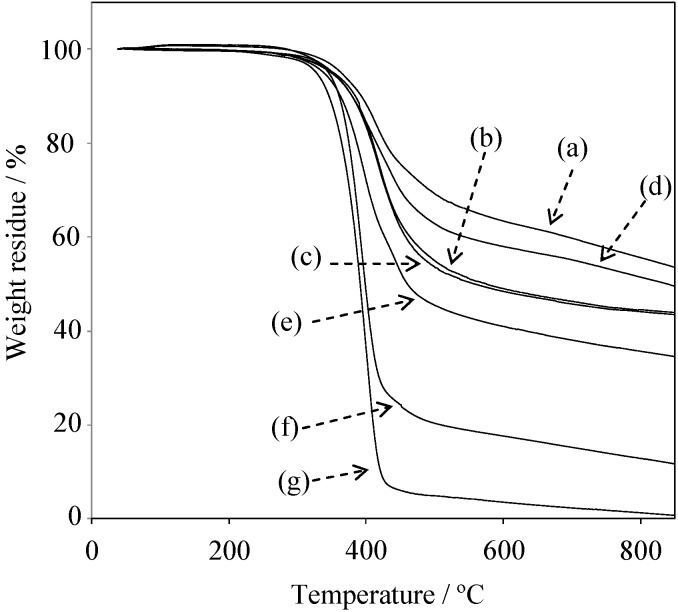
TGA curves of P(oligo(P-4va)) (a) and P(P-4va/BuA)s with 14 (b), 33 (c), 50 (d), 58 (e), and 63 (f) mol % of BuA composition.

Figure 14 shows the TGA results of the cured films of P(P-4va/BuA)s with various BuA compositions. The *T*_5_ and *T*_10_ of PBuA were 328 °C and 346 °C, respectively, indicating that PBuA also decomposed at the same temperature range of PSt and PMMA. Nevertheless, the presence of 50 mol % of BuA unit in PBZ, P(P-4va/BuA) indicated *T*_5_ and *T*_10_ at 352 and 380 °C, respectively, with the char yield at 850 °C was 49%. As summarized in Table 2, the cured P(P-4va/BuA) films showed high thermal stability over a wide composition range.

## 3. Experimental Section

### 3.1. Reagents

Phenol (99%) was purchased from Sigma-Aldrich Co. LLC (St. Louis, MO, USA). Paraformaldehyde (94%) and 4-vinylaniline (95%) were obtained from Tokyo Chemical Industry Co., LTD., Tokyo, Japan (TCI). Tetrahydrofuran (THF, 99.5%), benzene (99.5%), and chloroform (99%) were received from Wako Pure Chemical Industries, Ltd., Wako, Japan. The aforementioned reagents were used as received. 2,2'-Azobisisobutyronitrile (AIBN, 98%), obtained from TCI, was purified by recrystallization with MeOH. Styrene (St, >99%, Wako), methyl methacrylate (MMA, >98%, Wako), and *n*-butyl acrylate (BuA, >99%, TCI) were repeatedly washed with aqueous NaOH, and with distilled water, and then purified by fractional distillation under reduced nitrogen pressure, dried over CaH_2_, and vacuum-distilled just prior to use. Monofunctional BZ having vinyl group, P-4va, was synthesized according to the previous paper as a pale pink powder in 53% yield [39]. The thermal cure of P-4va was also performed as described in the previous paper [39].

### 3.2. Radical Polymerization of P-4va and Vinyl Monomers in the Presence of AIBN

Under nitrogen atmosphere, AIBN (16.4 mg, 0.1 mmol) was dissolved in benzene (2 mL), followed by adding P-4va (2.40 g, 10 mmol). The solution was degassed by freeze-thawing, and then the radical polymerization was carried out at 60 °C for 24 h. The polymerization was terminated by cooling to 0 °C. The reaction mixture was concentrated under reduced pressure at room temperature, and was then poured into large amount of MeOH. The precipitate was collected by filtration, and dried under vacuum to give oligo(P-4va). 

In the same manner, radical polymerization of vinyl monomers, St, MMA, and BuA was performed. To a benzene solution (2 mL) of AIBN (16.4 mg, 0.1 mmol) was added 10 mmol of the monomer. Each product was poured into large amount of MeOH, filtered, and dried under vacuum, affording polystyrene and PMMA as white powder. PBuA was obtained as while colorless sticky material. 

### 3.3. Radical Copolymerization of P-4va with Vinyl Monomers 

Similar to the radical polymerization of P-4va, under nitrogen atmosphere, AIBN (16.4 mg, 0.1 mmol) was dissolved in benzene (2 mL), followed by adding P-4va and co-monomer (St, MMA, or BuA, total monomer concentration: 5 mol/L). The solutions were degassed by freeze-thawing. The radical co-polymerization was carried out at 60 °C. After 24 h, the polymerization was terminated by cooling down to 0 °C. The products were concentrated under reduced pressure at room temperature, and then poured into large amount of MeOH. The brown precipitate was collected by filtration, and was dried under vacuum to give the copolymers. 

### 3.4. Thermal Cure of Oligo(P-4va) and Copolymer Films

Oligo(P-4va) and copolymers were dissolved in THF to prepare 30 wt % solutions by stirring at 80 °C for 3 h. The obtained reddish brown solutions were cast on glass substrates. The solvent was removed in air-oven at 50 °C for 12 h. The self-standing oligo(P-4va) and copolymer films were further cured at 100, 150, 200 and 240 °C for 1 h each, affording reddish transparent PBZ films. The thickness of the films was in the range of 45–75 μm.

### 3.5. Measurements 

NMR spectra were measured on a Mercury 300 spectrometer (300 MHz for ^1^H, Varian, Palo Alto, CA, USA) or a JNM-ECS400 spectrometer (400 MHz for ^1^H, JEOL, Tokyo, Japan). IR spectra were recorded on a model FT/IR-420 spectrophotometer (JASCO, Tokyo, Japan). Size exclusion chromatography (SEC) was measured with JASCO instrument with UV-2075 (254 nm) or refractive index detectors. Chloroform was used as the eluent at a flow rate of 1.0 mL/min at 40 °C. Two polystyrene gel columns (TSK-GEL MULTIPORE HLX-M) were used. Standard PMMA was used as the reference. Differential scanning calorimetry (DSC) was performed on a Thermo Plus 2 DSC8230 (Rigaku, Tokyo, Japan) at a heating rate of 10 °C/min under nitrogen. Thermogravimetric analysis (TGA) was measured on a Rigaku Thermo Plus 2 TG-DTA TG8120 at a heating rate of 5 °C/min under argon. Dynamic viscoelastic measurements were conducted on a model DDV-01FP Rheovibron automatic dynamic viscoelastomer (ORIENTEC, Tokyo, Japan) at 35 Hz at a heating rate of 4 °C/min.

## 4. Conclusions

Radical polymerization of the P-4va with AIBN gave s linear oligomer having BZ units as pendant groups. Radical copolymerization of P-4va with various vinyl monomers afforded a series of copolymers having pendent BZs, showing that BZ units were introduced easily into various types of vinyl polymers. The thermal curing of the oligomer or copolymers up to 240 °C gave self-standing films by the ring-opening polymerization of the pendent BZ groups. Although the cured films of oligo(P-4va) and P-4va/St copolymer were brittle, the cured film of P-4va/MMA copolymer with 17 mol % of MMA gave a tough film with enhanced thermal and mechanical properties compared with PBZ. Moreover, the cured film from P-4va/BuA copolymers with wide range of BuA compositiona exhibited significantly improved flexibility and the toughness of PBZ without PBuA segments. These results indicate that the synthesized copolymers containing pendent BZs can serve as high performance PBZs.

## Supplementary Materials

Supplementary materials can be accessed at: http://www.mdpi.com/1420-3049/20/04/6488/s1.
