# Supplementary material for: Copolymers of Vinyl-Containing Benzoxazine with Vinyl Monomers as Precursors for High Performance Thermosets
Source: Molecules. 2015 Apr 10;20(4):6488–503. doi: 10.3390/molecules20046488 (PMC6272515; doi:10.3390/molecules20046488)
Supplement: Supplementary file 1 [file molecules-20-06488-s001.pdf]

Supporting Information

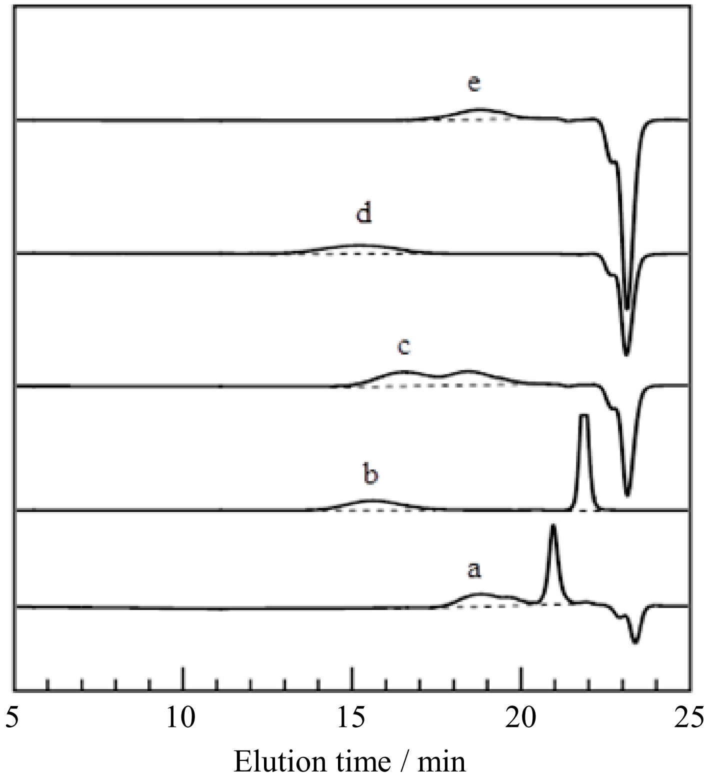

Figure S1. SEC curves of oligo(P-4va) (a), PSt (b), P(P-4va/St) (c), PMMA (d), and P(P-4va/MMA) (e).

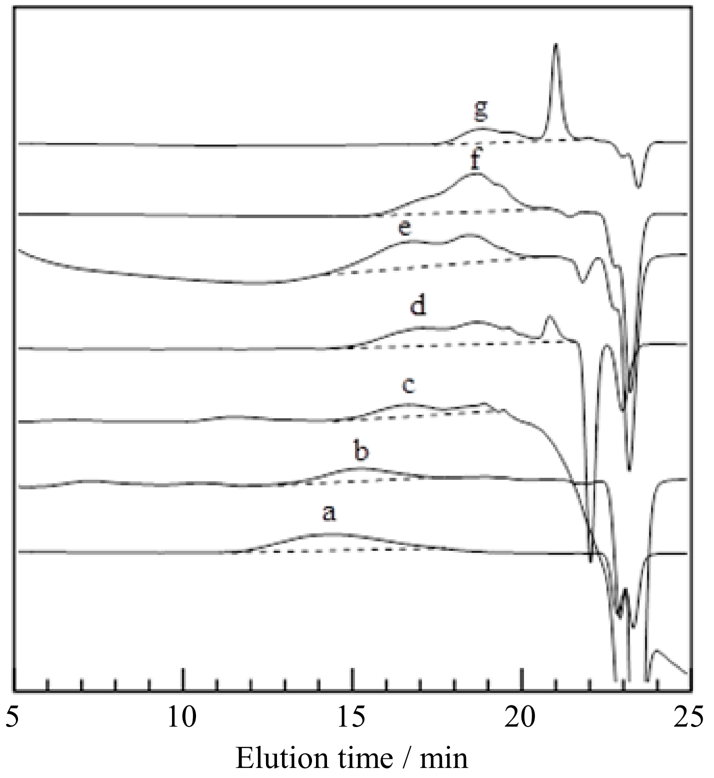

Figure S2. SEC curves of PBuA (a), P(P-4va/BuA) with 63 (b), 58 (c), 50 (d), 33 (e), and 14 (f) mol % of BuA composition, and oligo(P-4va) (g).
